# Hydrogen Sulfide Attenuated Sepsis-Induced Myocardial Dysfunction Through TLR4 Pathway and Endoplasmic Reticulum Stress

**DOI:** 10.3389/fphys.2021.653601

**Published:** 2021-06-09

**Authors:** Yu-hong Chen, Xu Teng, Zhen-jie Hu, Dan-yang Tian, Sheng Jin, Yu-ming Wu

**Affiliations:** ^1^Department of Physiology, Hebei Medical University, Shijiazhuang, China; ^2^Department of Critical Care Medicine, The Fourth Hospital of Hebei Medical University, Shijiazhuang, China; ^3^Hebei Collaborative Innovation Center for Cardio-Cerebrovascular Disease, Shijiazhuang, China; ^4^Key Laboratory of Vascular Medicine of Hebei Province, Shijiazhuang, China

**Keywords:** sepsis, myocardial dysfunction, hydrogen sulfide, Toll-like receptor 4, endoplasmic reticulum stress

## Abstract

**Aims:** We examined the change in endogenous hydrogen sulfide (H_2_S) production and its role in sepsis-induced myocardial dysfunction (SIMD).

**Results:** Significant elevations in plasma cardiac troponin I (cTnI), creatine kinase (CK), tumor necrosis factor-α (TNF-α), and interleukin-1β (IL-1β) were noted in SIMD patients, whereas left ventricular ejection fraction (LVEF), left ventricular fractional shortening (LVFS), and plasma H_2_S were significantly decreased relative to those in the controls. Plasma H_2_S was linearly related to LVEF and LVFS. Subsequently, an SIMD model was developed in mice by injecting lipopolysaccharide (LPS), and NaHS, an H_2_S donor, was used to elucidate the pathophysiological role of H_2_S. The mice showed decreased ventricular function and increased levels of TNF-α, IL-1β, cTnI, and CK after LPS injections. Toll-like receptor (TLR) 4 protein and endoplasmic reticulum stress (ERS) proteins were over expressed in the SIMD mice. All of the parameters above showed more noticeable variations in cystathionine γ-lyase knockout mice relative to those in wild type mice. The administration of NaHS could improve ventricular function and attenuate inflammation and ERS in the heart.

**Conclusion:** Overall, these findings indicated that endogenous H_2_S deficiency contributed to SIMD and exogenous H_2_S ameliorated sepsis-induced myocardial dysfunction by suppressing inflammation and ERS via inhibition of the TLR4 pathway.

## Introduction

Sepsis is the predisposing factor of organ failure and has a considerable mortality rate in critical care patients (Singer et al., 2016). The heart, as a circulatory system component, is a key target organ for injury in sepsis. Sepsis-induced myocardial dysfunction (SIMD) was observed in approximately half of the patients with sepsis, and associated with adverse outcomes and increased mortality (Frencken et al., 2018).

The etiology and pathogenesis of SIMD, including inflammation, altered metabolism, microcirculatory impairment, oxidative stress, and cell dysfunction, are incompletely understood (Liu et al., 2017). Inflammation is fundamental to the development of SIMD. Toll-like receptors (TLRs), particularly TLR4, increase inflammatory cytokines expression, especially tumor necrosis factor-α (TNF-α), interleukin-1β (IL-1β), and interleukin-6 (IL-6), which may lead to myocardial depression. Several studies have confirmed that TNF-α and IL-1β were associated with myocardial injury, and inhibition of TLR4 improved myocardial dysfunction (Zhao et al., 2016; Tan et al., 2019).

The endoplasmic reticulum (ER) is a system of tubular and flat vesicular structures for calcium storage. During inflammation, ischemia, stress, and other pathological states, misfolded/unfolded proteins aggregate in the ER and induce endoplasmic reticulum stress (ERS) (Wang et al., 2018). There are three ER-localized protein sensors for ERS: double-stranded RNA-dependent protein kinase RNA-like endoplasmic reticulum kinase (PERK), inositol-requiring enzyme 1α (IRE-1α), and activating transcription factor 6 (ATF6). In addition, glucose-regulated protein 78 (GRP78), CCAAT/enhancer-binding protein- homologous protein (CHOP), and caspase-12 are commonly used as ERS markers (Almanza et al., 2019). Recent studies have reported that ERS was involved in organ damage in septic animals and that inhibiting ERS could depress inflammation, attenuate organ damage, and improve survival (Liu et al., 2016; Zhong et al., 2019).

Hydrogen sulfide (H_2_S) is the most recently discovered gasotransmitter that is endogenously generated from cystathionine β-synthase (CBS), cystathionine γ-lyase (CSE), and 3-mercaptopyruvate sulfur transferase (3-MST) (Aroca et al., 2020). Earlier studies focused on the protective role of H_2_S during hypertension, stroke, and ischemia–reperfusion injury in multiple organ systems (Cui et al., 2020; Xia et al., 2020). Our team has previously shown that H_2_S was considered protective in takotsubo (stress-induced) cardiomyopathy and acute kidney injury by attenuating inflammation and stress-induced oxidative stress (Zhang Z. et al., 2017; Chen et al., 2018). However, it is not yet clear if H_2_S is involved in SIMD.

The study was aim to examine the change in H_2_S production and its role in SIMD.

## Materials and Methods

### Patients

Ten patients without sepsis as the controls and 10 patients with SIMD were recruited from the intensive care unit (ICU) of the Fourth Hospital of Hebei Medical University between July 2017 and December 2017. The inclusion criteria were (1) age > 18 years, (2) ICU stay > 48 h, and (3) SIMD patients who also fit the diagnosis of sepsis and SIMD (see below). Sepsis was defined according to consensus international guidelines (Sepsis-3.0) (Singer et al., 2016). When left ventricular ejection fraction (LVEF) deteriorated to < 50%, patients were defined as having SIMD (Cheng et al., 2019). The exclusion criteria were (1) heart surgery, heart attack, or acute exacerbation of previous heart disease in the last week; (2) previous abnormal echocardiography; and (3) pregnancy. Upon ICU admission, blood samples were collected. Ethics approval was obtained from the Research Ethics Committee of the Fourth Hospital of Hebei Medical University. All patients gave written informed consent. This study followed all of the applicable Chinese laws, regulations, and guidelines.

### Animals and Reagents

All experiments received Animal Care and Use Ethics Committee approval and conformed to the Guide for the Care and Treatment of Laboratory Animals (1985, NIH).

Male C57BL/6J 8–12-week-old mice were provided by Vital River Laboratories (Beijing, China). CSE heterozygous mice were a gift from Professor Yichun Zhu (Fudan University, Shanghai, China). The CSE heterozygous mice were used for reproduction, and CSE wild type (WT) and CSE knockout (CSE KO) mice were used for the formal trials. The mice were fed a normal diet under standard animal housing conditions.

LPS and NaHS (H_2_S donor) were purchased from Sigma (Sigma–Aldrich, United States), and NaHS was freshly prepared before each use. The cTnI, TNF-α, and IL-1β kits were obtained from Xinbosheng Bioengineering (Shenzhen, China). Bicinchoninic acid (BCA) reagent was obtained from Generay Biotechnology (Shanghai, China). All other chemicals were reagent grade and used as received.

### Animal Models and Groups

C57BL/6J mice were randomized into three groups: the Control, LPS, and LPS + NaHS (50 μmol/kg) groups (*n* = 8 in each group). The WT and CSE KO mice were arranged into five groups: the WTControl, WT + LPS, CSE KOControl, CSE KO + LPS, and CSE KO + LPS + NaHS (100 μmol/kg) groups (*n* = 8 in each group).

The LPS (10 mg/kg) administration method was an established model for SIMD (Ndongson-Dongmo et al., 2015), and NaHS was administered intraperitoneally 3 h after LPS treatment. Blood samples and heart tissues were obtained 6 h after LPS treatment. After centrifugation for 10 min at 4,000 rpm at4°C, plasma was removed and frozen at –80°C. The hearts were fixed in 4% paraformaldehyde, and left ventricle tissues were frozen at –80°C.

### Measurement of Echocardiography

We used a Vevo 2100 ultrasound device (Visual Sonics Inc., Toronto, Canada) to obtain transthoracic M-mode echocardiography 6 h after LPS treatment. Five consecutive cardiac cycles were captured, LVEF and LVFS were measured, and average readings were recorded.

### Histological Analysis

The heart samples were routinely fixed with paraformaldehyde (4%) for 48 h and mounted with paraffin. Five-μm sections were stained with hematoxylin and eosin (HE) and observed under a light microscope for histopathological analysis (Olympus BX40, Tokyo, Japan). Histological changes were assessed via quantitative measurements of tissue damage by a blinded observer. Pathology scores were assigned as follows (Kishimoto et al., 2001): 0 = normal; 1 = lesions < 25% of the myocardium; 2 = lesions between 25 and 50% of the myocardium; 3 = lesions between 50 and 75% of the myocardium; and 4 = lesions between 75 and 100% of the myocardium.

### Measurement of CK, cTnI, and Inflammatory Cytokines in Plasma

Plasma CK level was tested by using an automatic biochemical analyzer (Cobas 6000, Roche, Switzerland). Enzyme-linked immunosorbent assay (ELISA) kits (Xinbosheng, Shenzhen, China) were used to determine cTnI, TNF-α, and IL-1β levels in plasma following the manufacturer instructions.

### Measurement of H_2_S Levels

H_2_S concentrations in the plasma and hearts were measured as previously described with some modifications (Tan et al., 2017). First, snap-frozen cardiac tissue was homogenized and centrifuged (12,000 rpm, 20min, 4°C). The protein content in supernatants was measured by using the BCA reagent. Plasma was thawed and mixed before assay. Thirty microliter of samples (plasma or supernatant) was spiked with 80μL monobromobimane (Sigma–Aldrich) and 10 μL of 0.1% ammonia. After being agitated for 60 min at room temperature, the reaction was stopped by adding 10 μL of 20% formic acid. Then, the mixture was subjected to centrifugation at 15,000rpm for 10min, clear supernatants were collected as samples. H_2_S levels were quantified against a standard curve generated by Na_2_S (0–40 μmol/L). The plasma H_2_S levels were expressed as μmol/L, and the heart H_2_S levels were divided by the protein concentrations, which were expressed as μmol/g of protein.

### Western Blot Analysis

Heart tissues were homogenized in lysis buffer, and protein content was measured by using the BCA reagent. Equivalent amounts of protein were separated in 10% sodium dodecyl sulfate–polyacrylamide gel electrophoresis and transferred to polyvinylidene fluoride membranes (Millipore-Upstate). Membranes were blocked with 5% non-fat milk for 1 h and incubated overnight at 4°C in primary antibodies, anti-CSE (1:1,000, Proteintech, United States), anti-CBS (1:1,000, Proteintech, United States), anti-3-MST (1:500, Abcam, United States), anti-TLR4 (1:500, Abcam, United States), anti-CHOP (C/EBP homologous protein) (1:1,000, Abcam, United States), anti-GRP78 (78-kDa glucose-regulated protein) (1:1,000, Abcam, United States), anti-caspase-12 (1:1,000, Abcam, United States), anti-total PERK (1:1,000, Santa Cruz, United States), anti-pPERK (1:1,000, Santa Cruz, United States), anti-ATF6 (1:1,000, Santa Cruz, United States), anti-IRE-1α (1:1,000, Abcam, United States), anti-pIRE-1α (1:1,000, Abcam, United States), and β-actin (1:1,000, Proteintech, United States). The membranes were rinsed in tris-buffered saline and Tween 20 three times for 5 min and incubated with horseradish-peroxidase-conjugated secondary antibodies for 1 h. Specific bands were developed by using a chemiluminescence detection system (Thermo, Scientific-Pierce). The band intensity was quantified by Image J software.

### Statistical Analysis

All data were analyzed by using SPSS version 21.0 statistical software (IBM SPSS Statistics for Windows, IBM Corp., Armonk, NY). Data of patients were presented as the mean ± deviation (SD). Data of mice were presented as the mean ± standard error of the mean (SEM). Student’s *t*-test was used to assess the difference between two groups, whereas one-way analysis of variance (ANOVA) was used for analysis for three or more groups. The least significant difference method was used after ANOVA to determine differences between groups. Statistical significance was defined as *P* < 0.05.

## Results

### Plasma H_2_S Level Decreased in SIMD Patients

**FIGURE 1:**
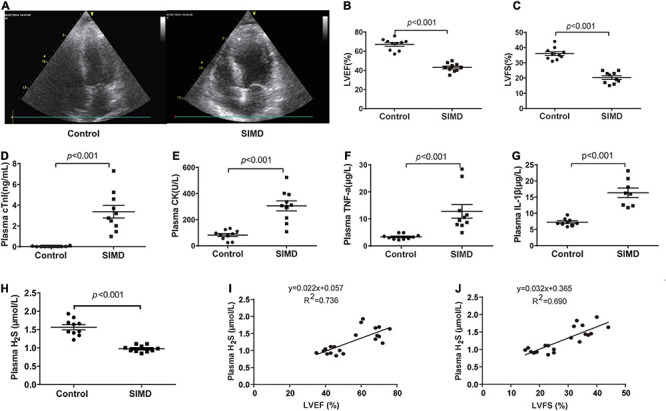
Plasma H_2_S level decreased in SIMD patients: **(A)** Representative echocardiogram images from the Control group and the SIMD group. **(B)** The changes of left ventricular ejection fraction (LVEF). **(C)** The changes of left ventricular fractional shortening (LVFS). **(D)** cTnI levels in the plasma. **(E)** CK levels in the plasma. **(F)** TNF-α levels in the plasma. **(G)** IL-1β levelsin the plasma. **(H)** H_2_S levels in the plasma. **(I,J)** Plasma H_2_S level is positively correlated with LVEF and LVFS. *n* = 10 in every group. Results are means ± SD. *p* < 0.05 was considered significant.

To investigate H_2_S level in SIMD patients, we used a cohort of 10 controls and 10 patients with SIMD whose characteristics were shown in Table 1. There were no differences between the two groups in age, gender, body mass index (BMI) and previous history of chronic heart disease. The LVEF and LVFS levels were lower in the SIMD group than in the Control group (Figures 1A–C). The cardiac troponin I (cTnI), creatine kinase (CK), TNF-α, and IL-1β levels in plasma were significantly higher in the SIMD patients than in the controls (Figures 1D–G, respectively). The plasma H_2_S level was significantly lower in the SIMD group than in the Control group (Figure 1H), and plasma H_2_S showed linear relationships with LVEF and LVFS (Figures 1I,J).

**TABLE 1:** Characteristics of control and SIMD patients.

	**Control (*n* = 10)**	**SIMD (*n* = 10)**	***p***
Age, yrs, mean [SD]	70.3 [8.8]	68.9 [14.8]	NS
Gender, male (%)	5 (50)	5 (50)	NS
BMI, Kg/m^2^, mean [SD]	24.1 [1.6]	22.9 [2.0]	NS
Etiology of sepsis, byorgan (n)	NA	Respiratory (3) Abdominal (4) Genitourinary (2) Intestines(1)	
Previous history of chronic heart disease (yes/no)	0/10	0/10	NS
T, °C, mean [SD]	36.4 [0.2]	38.6 [0.5]	0.018
HR, beats/min, mean [SD]	75.6 [7.4]	101.5 [15.7]	0.018
RR, times/min, mean [SD]	14.0 [1.5]	23.5 [5.5]	0.007
WBC, ×10^9^/L, mean [SD]	5.9[1.3]	14.8[4.0]	<0.001
LVEF, %, mean [SD]	75.1[6.5]	42.0 [5.5]	<0.001

**BMI, body mass index; T, temperature; HR, heart rate; RR, breathing rate; WBC, White blood cell; LVEF, left ventricular ejection fraction; NS, not significant; NA, not applicable.**

### Loss of Endogenous H_2_S Aggravated LPS-Induced Myocardial Dysfunction

**FIGURE 2:**
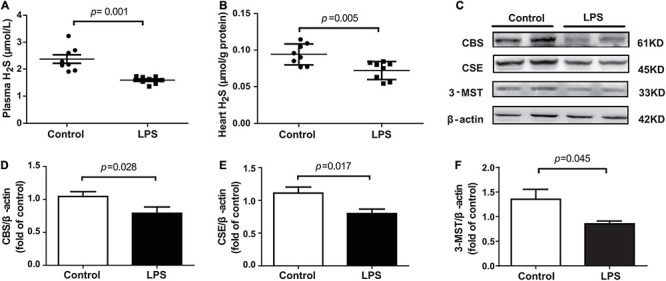
LPS decreased plasma and heart tissues levels of H_2_S in LPS-induced myocardial dysfunction mice: **(A)** H_2_S levels in the plasma. **(B)** H_2_S levels in the heart tissues. **(C–F)** Representative Western blots and quantification of CBS, CSE, and 3-MST protein expression in heart tissues. β-actin was used as the internal control. *n* = 8 in every group. Results are means ± SEM. *p* < 0.05 was considered significant.

**FIGURE 3:**
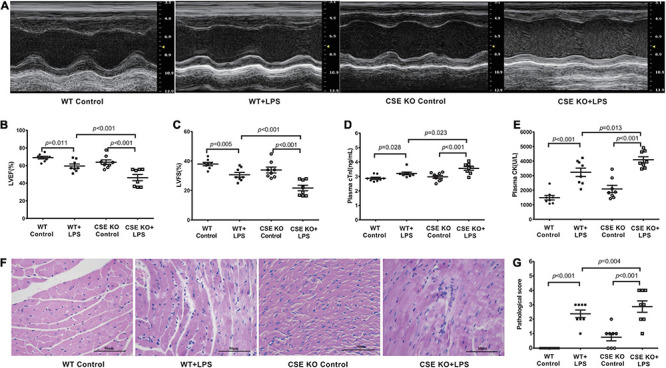
Loss of endogenous H_2_S aggravated LPS-induced myocardial dysfunction: **(A)** Representative M-mode images from WT Control, WT + LPS, CSE KO Control, and CSE KO + LPS groups. **(B)** The changes of left ventricular ejection fraction (LVEF). **(C)** The changes of left ventricular fractional shortening (LVFS). **(D)** cTnI levels in the plasma. **(E)** CK levels in the plasma. **(F)** Representative HE-stained left ventricular sections (scale bar = 50μm). **(G)** Pathological score of the HE-stained left ventricular sections. *n* = 8 in every group. Results are means ± SEM. *p* < 0.05 was considered significant.

The levels of H_2_S in the plasma and heart tissue in mice were significantly decreased after LPS injection (Figures 2A,B). H_2_S is generated by CBS, CSE, and 3-MST, so we examined the expressions of three enzymes in the hearts of mice concomitantly. Our results showed that the expressions of these three enzymes, especially of CSE, were significantly lower in the LPS-treated mice than in the controls (Figures 2C–F). CSE was the main H_2_S-producing enzyme in the cardiovascular system, so CSE KO mice, which had lower H_2_S levels in plasma and heart (Supplementary Figures 1A–C), were used to determine whether endogenous H_2_S was involved in the progression of SIMD. Our results showed that the levels of LVEF and LVFS were significantly decreased (Figures 3A–C), whereas cTnI and CK levels were significantly higher (Figures 3D,E) in the WT + LPS group than in the WT Control group. HE staining showed a normal morphology of hearts from the controls, whereas the morphology of those in the LPS group exhibited the characteristic findings of acute cardiomyocyte injury and inflammation, including tissue edema, inflammatory cell infiltration, and nuclear swelling (Figures 3F,G). A similar result was observed between the CSE KO Control group and CSE KO + LPS group, but the mice in the CSE KO + LPS group had lower cardiac function and more severe myocardial injury than those in the WT + LPS group (Figures 3A–G).

### Exogenous H_2_SAmeliorated LPS-induced Myocardial Dysfunction

**FIGURE 4:**
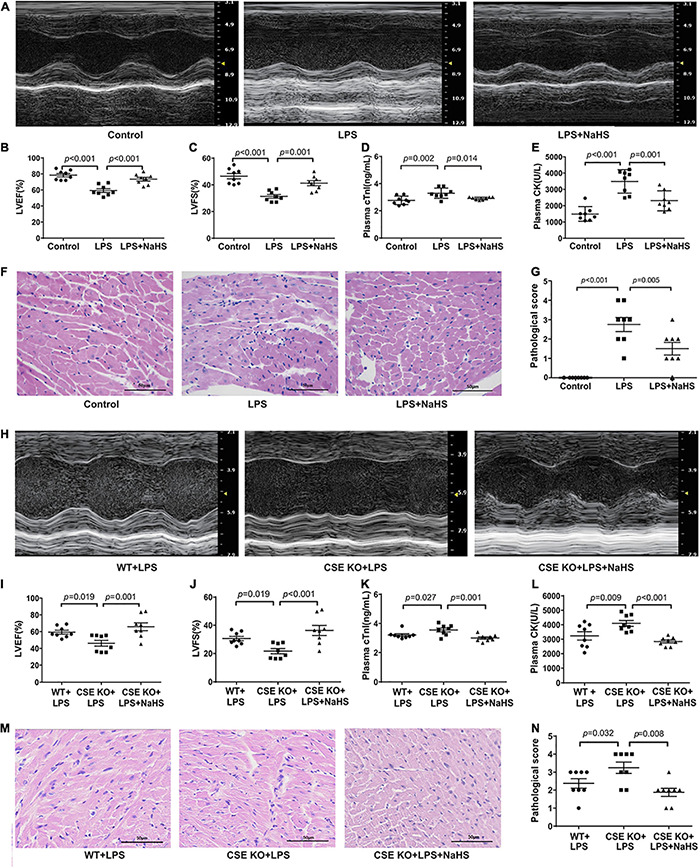
Exogenous H_2_S ameliorated LPS-induced myocardial dysfunction: **(A)** Representative M-mode images from the Control, LPS, and LPS + NaHS groups. **(B)** The changes of left ventricular ejection fraction (LVEF). **(C)** The changes of left ventricular fractional shortening (LVFS). **(D)** cTnI levels in the plasma. **(E)** CK levels in the plasma. **(F)** Representative HE-stained left ventricular sections (scale bar = 50 μm). **(G)** Pathological score of the HE-stained left ventricular sections. **(H)** Representative M-mode images from the WT + LPS, CSE KO + LPS, and CSE KO + LPS + NaHS groups. **(I)** The changes of left ventricular ejection fraction (LVEF). **(J)** The changes of left ventricular fractional shortening (LVFS). **(K)** cTnI levels in the plasma. **(L)** CK levels in the plasma. **(M)** Representative HE-stained left ventricular sections (scale bar = 50 μm). **(N)** Pathological score of the HE-stained left ventricular sections. *n* = 8 in every group. Results are means ± SEM. *p* < 0.05 was considered significant.

C57BL/6J mice were intraperitoneally injected with NaHS at doses of 10 μmol/kg, 50 μmol/kg, or 100 μmol/kg (*n* = 8) 3 h after the injection of LPS. A 50 μmol/kg dose of NaHS improved the cardiac function of LPS-treated mice (Supplementary Figures 2A,B), so the dose of 50 μmol/kg was chosen for subsequent experiments. Treatment with 50 μmol/kg NaHS significantly improved myocardial dysfunction and reduced the plasma levels of cTnI and CK (Figures 4A–E). HE staining in the LPS + NaHS groupalso showed less cardiomyocyte injury and inflammation (Figures 4F,G).

A 50 μmol/kg dose of NaHS did not improve heart function in the CSE KO + LPS group, and a higher dose of 100 μmol/kg was needed (Supplementary Figures 2C,D). Treatment with 100 μmol/kg NaHS significantly improved the myocardial dysfunction (Figures 4H–J) and reduced the plasma levels of cTnI and CK (Figures 4K,L) of the mice in the CSE KO + LPS + NaHS group. Histological examination showed that the cardiomyocyte injury was more severe in the CSE KO + LPS group than in the WT + LPS group, and treatment with 100 μmol/kg NaHS significantly attenuated the damage (Figures 4M,N).

### H_2_S Inhibited Inflammation and ERS in LPS-Induced Myocardial Dysfunction

**FIGURE 5:**
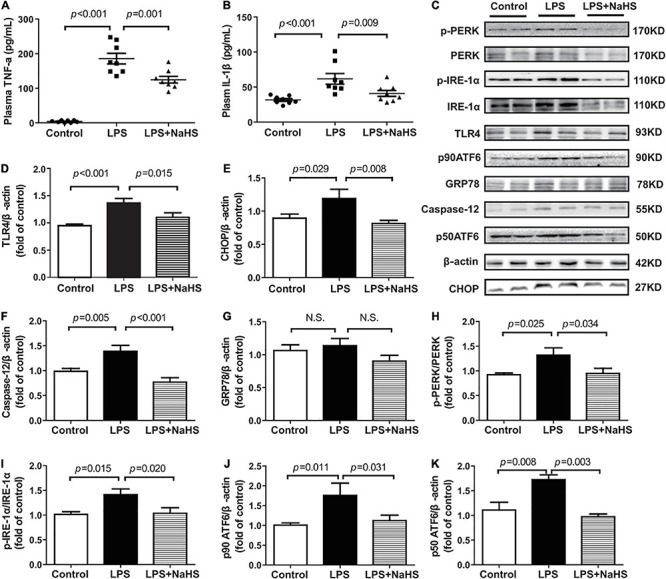
H_2_S inhibited inflammation and ERS in LPS-induced myocardial dysfunction: **(A)** TNF-α levels in the plasma. **(B)** IL-1β levels in the plasma. **(C–K)** Representative Western blots and quantitative analysis for TLR4, CHOP, Caspase-12, GRP78, p-PERK/PERK, p-IRE1/IRE1, p90 ATF6, and p50 ATF6 protein expression in heart tissues. β-actin was used as the internal control. *n* = 8 in every group. Results are means ± SEM. *p* < 0.05 was considered significant.

We investigated the contribution of exogenous H_2_S to inflammation in mice with SIMD. Plasma TNF-α and IL-1β and TLR4 expressions in the heart were obviously higher in the LPS group than in the Control group. Additionally, treatment with 50 μmol/kg NaHS significantly decreased the TNF-α and IL-1β levels as well as the expression level of TLR4 (Figures 5A,B,D). The effects of exogenous H_2_S on the expression of ERS-related proteins in the heart specimens were also evaluated by western blot analysis and the representative western blots were shown in Figure 5C. PERK, IRE-1, and ATF6 are the primary effectors of ERS. GRP78 relocates from the luminal domains of PERK, IRE-1, and ATF6 and contributes to activation of these three effectors, which can activate the downstream transcription factors (CHOP and caspase-12) and induce inflammatory responses. We found that the expressions of CHOP, caspase-12, PERK, ATF6, and IRE-1α were obviously higher in the LPS group than in the Control group, which were significantly reduced by subsequent treatment with 50 μmol/kg NaHS (Figures 5E,F,H–K). However, there were no significant differences in GRP78 expression among the three groups (Figure 5G).

**FIGURE 6:**
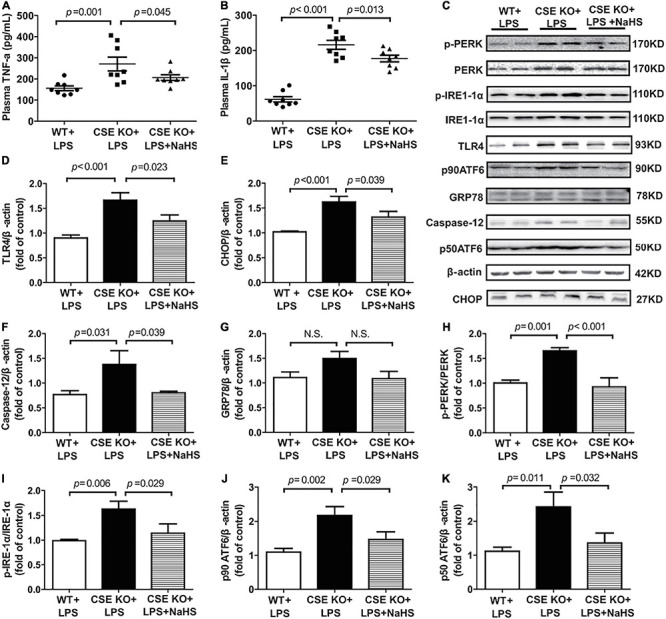
H_2_S inhibited inflammation and ERS in CSE KO mice: **(A)** TNF-α levels in the plasma. **(B)** IL-1β levels in the plasma. **(C–K)** Representative Western blots and quantitative analysis for TLR4, CHOP, Caspase-12, GRP78, p-PERK/PERK, p-IRE1/IRE1, p90 ATF6, and p50 ATF6 protein expression in heart tissues. β-actin was used as the internal control. *n* = 8 in every group. Results are means ± SEM. *p* < 0.05 was considered significant.

The effect of endogenous H_2_S on inflammation and ERS was also assessed and the representative western blots were shown in Figure 6C. The levels of TNF-α and IL-1β and the expression of TLR4 were obviously higher in the CSE KO + LPS group than in the WT + LPS group, and treatment with 100 μmol/kg NaHS significantly decreased the TNF-α and IL-1β levels and TLR4 expression (Figures 6A,B,D). The protein expressions of CHOP, caspase-12, PERK, ATF6, and IRE1-α were obviously higher in the CSE KO + LPS group than those in the WT + LPS group. Treatment with 100 μmol/kg NaHS significantly inhibited CHOP, caspase-12, PERK, ATF6, and IRE1-α expressions (Figures 6E,F,H–K). However, there were no significant differences in GRP78 expression among the three groups (Figure 6G).

## Discussion

Our results revealed three important findings. First, endogenous H_2_S was significantly decreased in the patients with SIMD. Second, H_2_S had a protective role in SIMD. Third, inhibition of inflammation and ERS might mediate the protective effect of H_2_S against SIMD.

Sepsis is a systemic inflammation induced by infection, and severe sepsis is the main cause of high mortality (Coopersmith et al., 2018). SIMD is a transient cardiac dysfunction that usually results in hemodynamic instability, which is generally believed to be the cause of death for patients with sepsis (L’Heureux et al., 2020). In 1984, Parker and Parrillo et al. first introduced the definition of SIMD, including decreased LVEF and increased end-diastolic volume index (Parker et al., 1984). However, the mechanism of SIMD is not certain, and inflammation is believed to be one of the pathological bases of cardiac injury in sepsis (L’Heureux et al., 2020). During sepsis, pathogen-associated molecular patterns and damage-associated molecular patterns activate TLRs, which then trigger inflammation by stimulating the production and release of pro-inflammatory cytokines, specifically TNF-α and IL-1β (Yang et al., 2018). Tang et al. (2014) demonstrated that TNF-α expression in cardiomyocytes was increased after dealing with LPS, and propofol alleviated cardiac dysfunction by decreasing the generation of TNF-α. Hobai et al. (2015) reported that the contractility of adult rat ventricular myocytes could be depressed after long-term exposure to LPS and an inflammatory cytokines mixture (TNF, IL-1, and IL-6). Previous studies confirmed that cTnI was a highly sensitive and specific marker of SIMD, as well as an independent risk factor for mortality (Liu et al., 2017; L’Heureux et al., 2020). In our study, patients with SIMD were defined as having an LVEF of < 50%, and the levels of cTnI, CK, TNF-α, and IL-1β in plasma were increased. We successfully replicated the SIMD model by using LPS in mice, and the results were in agreement with our results in humans and the results of previous studies (Li et al., 2019; Huang et al., 2020).

H_2_S, a gaseous mediator along with NO and CO, is a pivotal factor in modulating cardiovascular function. Previous studies have confirmed the cardiac protective effect of H_2_S, however, the specific mechanisms remain unclear in sepsis (Li et al., 2017; Zhang H. X. et al., 2017; Liu et al., 2019). It was reported that the lower plasma level of H_2_S indirectly predicted increased morbidity and mortality (Perridon et al., 2016; Yuan et al., 2017). In our study, we noticed that plasma H_2_S level decreased in SIMD patients and mice, and H_2_S levels in hearts of SIMD mice also decreased. We further evaluated the expressions of H_2_S-producing enzymes in the hearts of mice, and found that LPS could reduce the expressions of CBS, CSE, and 3-MST, which may be mainly responsible for endogenous H_2_S deficiency. Because CSE was the main H_2_S-producing enzyme in the heart, CSE KO mice which had lower H_2_S levels in plasma and heart were used to investigate the role of endogenous H_2_S in SIMD mice. The results showed that cardiac function was worse in the CSE KO + LPS group than those in the WT + LPS group. The plasma levels of TNF-α, IL-1β, cTnI, and CK were higher in the CSE KO + LPS group than those in the WT + LPS group. These data were consistent with those of a recently published study showing that H_2_S level in the heart and the survival rate were lower in CSE KO mice with myocardial infarction than in WT mice (Miao et al., 2016). Our result suggested that H_2_S deficiency participated in the pathophysiology and progression of SIMD.

The role of hydrogen sulfide in inflammation remains controversial. Zhang et al. (2006) showed that plasma H_2_S significantly increased in mice that received CLP, and an intraperitoneal bolus of H_2_S (10 mg/kg) increased mortality. Conversely, Ferlito et al. (2014) reported that H_2_S improved the prognosis of sepsis when administered subcutaneously at a dose of 100 μmol/kg (5.6 mg/kg). Therefore, we investigated whether exogenous H_2_S could protect cardiac function from LPS-induced myocardial dysfunction *in vivo*. We injected NaHS at doses of 10, 50, and 100 μmol/kg and found that 50 μmol/kg and 100 μmol/kg improved the cardiac function of SIMD mice. However, there was no significant difference in the improvement effects between the two dosages, so we used 50 μmol/kg NaHS to further elucidate the underlying mechanisms. We found that 50 μmol/kg NaHS could not improve cardiac function in the CSE KO + LPS group, but improvement was observed after administering a dose of 100 μmol/kg NaHS. One explanation for this result might be that the H_2_S level in plasma was lower in the CSE KO mice than in the WT mice after LPS administration. These results once again indicated that deficiencies of endogenous H_2_S may exert a negative effect on cardiac function, and H_2_S supplementation may be protective in SIMD.

Previous studies have demonstrated that ERS proteins were involved in the pathophysiology of sepsis-associated organ damage, including SIMD, by tightly recycling cytoplasmic calcium concentrations (Pang et al., 2019; Zhong et al., 2019). Sodhi et al. (2015) explored the role of ERS in traumatic/hemorrhagic lung injury and showed that TLR4 activation in epithelial cells led to ERS. They also showed that TLR4 inhibitors reduced ERS and lung injury by inhibiting the TLR4 signaling pathway. Similarly, Hiramatsu et al. (2006) found that GRP78 expression in the spleen, lungs, kidneys, liver, and heart of septic mice were significantly increased after intravenous LPS administration, indicating that LPS-induced systematic ERS. Zhang et al. (2015) also demonstrated that the expressions of GRP94, CHOP, and caspase-12 in the hearts of septic rats were upregulated and cardiomyocytes apoptosis could be attenuated by inhibiting ERS. A review stated that the ERS-related genes GRP78, IRE-1α, PERK, ATF6, CHOP, and caspase-12 were all upregulated in sepsis (Khan et al., 2015). We found that LPS increased the expression of TLR4, an upstream receptor in the inflammatory signaling pathway, and the expression of ERS effectors (PERK, p-PERK, IRE-1α, p-IRE-1α, p90ATF6, and p50ATF6) and downstream transcription factors (CHOP and caspase-12), finally resulted in increased levels of TNF-α and IL-1β in plasma. The expressions of the above markers were also higher in the CSEKO + LPS group than in the WT + LPS group, which was similar to the results of previous studies.

As a gasotransmitter, H_2_S has been recognized for its important role in various disease models by inhibiting ERS. In a renal ischemia–reperfusion model in rats, Ling et al. (2017) found that ERS-related signaling pathways (i.e., PERK, ATF6, and IRE1) and related proteins (GRP78, GRP94, XBP1, and eIF2α) were upregulated significantly in ischemia–reperfusion rats, with concomitant reduction in the levels of endogenous H_2_S. They also showed that NaHS treatment improved kidney damage by decreasing the expression of ERS-related genes. In mice with sepsis-associated organ damage, Ferlito et al. (2014) reported that CHOP expression was upregulated in the spleen, lungs, and liver, whereas exogenous H_2_S reduced the expression of CHOP in these organs, alleviated the inflammatory response, and improved the survival rate in septic mice, indicating that H_2_S had a protective role in sepsis by inhibiting ERS. Li et al. (2016) also examined the effect of H_2_S on the cardiac function of diabetic rats and reported that GRP78, CHOP, and caspase-12 expressions were elevated in the hearts of the diabetic rats relative to those of the controls. In that study, NaHS reduced the expression of the three ERS markers and improved myocardial hypertrophy and myocardial collagen deposition in diabetic rats, which demonstrated that H_2_S can attenuate cardiac dysfunction caused by diabetes by suppressing ERS. In the present study, we found that 50 and 100 μmol/kg NaHS downregulated the expressions of TLR4 and ERS markers in the LPS + NaHS group and CSE KO + LPS + NaHS group, respectively. The levels of TNF-α and IL-1β in plasma showed the same trend. Overall, our findings provided further evidence that H_2_S could reduce LPS-induced inflammation and ERS by inhibiting TLR4 and might have a cardioprotective role in SIMD.

This study also has several limitations. One limitation is the choice of H_2_S donors. There is still a lack of compounds that would meet all requirements for the ideal H_2_S donor in clinical studies. The different H_2_S releasing pattern of these donors may explain the difference of their effects in different models (Wu et al., 2016). SIMD is an acute disease process and a rapid releasing H_2_S donor is needed to elevate H_2_S concentration in the plasma as soon as possible. Previous studies from our group and others also showed that NaHS was often used in the acute inflammation models induced by LPS (Chen et al., 2018; Sun et al., 2019). Also, in our preliminary study, we found that NaHS could improve ventricular function of SIMD mice. However, NaHS salt will generate poly sulfides upon rapid oxidation in aqueous solution, which maybe not the best H_2_S donor. Another limitation to this study is the method to measure free hydrogen sulfide levels in blood or heart tissues. H_2_S exists as three forms in aqueous solution, including hydrogen sulfide gas, hydrosulfide anion (HS^–^), sulfide anion (S^2–^) and other bound forms. Hence it’s difficult to accurately measure H_2_S. Though various methods (such as colorimetry, gas chromatography, electrodes selective for sulfide, polarographic sensors, fluorescent probes, and high performance liquid chromatography) have been established, and the monobromobimane method we used is not specified (not just H_2_S, also include S^2–^ and HS^–^), but is one of the most widely used methods at present for simple and robust (Tan et al., 2017). The third limitation, we are not well-known of mechanisms about how LPS works in the reduction of H_2_S. Previous studies have implicated a role for PI3K/Akt/Sp1 signaling in the regulation of CSE (Yin et al., 2012), LPS may also influence CSE production through the same signaling.

## Conclusion

We showed that a deficiency in endogenous H_2_S contributed to SIMD, whereas exogenous H_2_S ameliorated SIMD by inhibiting inflammation and ERS via inhibition of the TLR4 pathway. These findings shed light on the role of H_2_S in treating SIMD.

## Data Availability Statement

The original contributions presented in the study are included in the article/Supplementary Material, further inquiries can be directed to the corresponding author/s.

## Ethics Statement

All our animal experimental procedures were performed according to the Guide for the Care and Use of Laboratory Animals of the National Institutes of Health (NIH) of the United States and approved by the Ethics Committee for Laboratory Animals Care and Use of Hebei Medical University.

## Author Contributions

Y-HC and Y-MW were involved in design. Y-HC and D-YT carried out the clinical and animal experiments. Y-HC and SJ summarized the data, performed analysis, and drafted the manuscript. Y-MW, XT, and Z-JH revised the final manuscript. All authors contributed to the article and approved the submitted version.

## Conflict of Interest

The authors declare that the research was conducted in the absence of any commercial or financial relationships that could be construed as a potential conflict of interest.

## Abbreviations

ATF6: Activating transcription factor 6
CHOP: CCAAT/enhancer-binding protein-homologous protein
CBS: Cystathionine β-synthase
CSE: Cystathionine γ-lyase
CK: Creatinekinase
H_2_S: Hydrogen sulfide
cTnI: Cardiac troponinI
CSE KO: CSE knockout
ER: Endoplasmic reticulum
ERS: Endoplasmic reticulum stress
GRP78: Glucose-regulated protein 78
IRE-1 α: Inositol-requiring enzyme 1 α
ICU: Intensive care unit
IL-1 β: Interleukin-1 β
PERK: Protein kinase RNA-like endoplasmic reticulum kinase
LPS: Lipopolysaccharide
TNF- α: Tumor necrosis factor- α
TLR: Toll-like receptor
LVEF: Left ventricular ejection fraction
LVFS: Left ventricular fractional shortening
SIMD: Sepsis-induced myocardial dysfunction
WT: Wild-type
3-MST: 3-mercaptopyruvate sulfur transferase.

## References

[B1] Almanza A. Carlesso A. Chintha C. Creedican S. Doultsinos D. Leuzzi B. (2019). Endoplasmic reticulum stress signalling - from basic mechanisms to clinical applications. *FEBS J.* 286 241–278. 30027602 10.1111/febs.14608 PMC7379631

[B2] Aroca A. Gotor C. Bassham D. C. Romero L. C. (2020). Hydrogen Sulfide: From a Toxic Molecule to a Key Molecule of Cell Life. *Antioxidants* 9:621. 10.3390/antiox9070621 32679888 PMC7402122

[B3] Chen Y. Jin S. Teng X. Hu Z. Zhang Z. Qiu X. (2018). Hydrogen Sulfide Attenuates LPS-Induced Acute Kidney Injury by Inhibiting Inflammation and Oxidative Stress. *Oxid. Med. Cell Longev.* 2018:6717212. 10.1155/2018/6717212 PMC5831990 29636853

[B4] Cheng M. M. W. Long Y. Wang H. Han M. M. W. Zhang J. Cui N. (2019). Role of the mTOR Signalling Pathway in Human Sepsis-Induced Myocardial Dysfunction. *Can. J. Cardiol.* 35 875–883. 10.1016/j.cjca.2019.03.022 31292086

[B5] Coopersmith C. M. De Backer D. Deutschman C. S. Ferrer R. Lat I. Machado F. R. (2018). Surviving sepsis campaign: research priorities for sepsis and septic shock. *Intensive Care Med.* 44 1400–1426. 29971592 10.1007/s00134-018-5175-z PMC7095388

[B6] Cui C. Fan J. Zeng Q. Cai J. Chen Y. Chen Z. (2020). CD4+ T-Cell Endogenous Cystathionine γ Lyase-Hydrogen Sulfide Attenuates Hypertension by Sulfhydrating Liver Kinase B1 to Promote T Regulatory Cell Differentiation and Proliferation. *Circulation* 142 1752–1769. 10.1161/circulationaha.119.045344 32900241

[B7] Ferlito M. Wang Q. Fulton W. B. Colombani P. M. Marchionni L. Fox-Talbot K. (2014). Hydrogen sulfide increases survival during sepsis: protective effect of CHOP inhibition. *J. Immunol.* 192 1806–1814. 10.4049/jimmunol.1300835 24403532 PMC3946246

[B8] Frencken J. F. Donker D. W. Spitoni C. Koster-Brouwer M. E. Soliman I. W. Ong D. S. Y. (2018). Myocardial Injury in Patients With Sepsis and Its Association With Long-Term Outcome. *Circ. Cardiovasc. Qual. Outcome* 11:e004040. 10.1161/CIRCOUTCOMES.117.004040 29378734

[B9] Hiramatsu N. Kasai A. Hayakawa K. Yao J. Kitamura M. (2006). Real-time detection and continuous monitoring of ER stress in vitro and in vivo by ES-TRAP: evidence for systemic, transient ER stress during endotoxemia. *Nucleic Acids Res.* 34:e93. 10.1093/nar/gkl515 16877567 PMC1540736

[B10] Hobai I. A. Morse J. C. Siwik D. A. Colucci W. S. (2015). Lipopolysaccharide and cytokines inhibit rat cardiomyocyte contractility in vitro. *J. Surg. Res.* 193 888–901. 10.1016/j.jss.2014.09.015 25439505 PMC4268427

[B11] Huang S. Xu M. Liu L. Yang J. Wang H. Wan C. (2020). Autophagy is involved in the protective effect of p21 on LPS-induced cardiac dysfunction. *Cell Death Dis.* 11:554. 10.1038/s41419-020-02765-7 PMC7374585 32694519

[B12] Khan M. M. Yang W. L. Wang P. (2015). Endoplasmicreticulum stress in sepsis. *Shock* 44 294–304. 26125088 10.1097/SHK.0000000000000425 PMC4575622

[B13] Kishimoto C. Kawamata H. Sakai S. Shinohara H. Ochiai H. (2001). Enhanced production of macrophage inflammatory protein 2 (MIP-2) by in vitro and in vivo infections with encephalomyocarditis virus and modulation of myocarditis with an antibody against MIP-2. *J. Virol.* 75 1294–1300. 10.1128/jvi.75.3.1294-1300.2001 11152502 PMC114035

[B14] L’Heureux M. Sternberg M. Brath L. Turlington J. Kashiouris M. G. (2020). Sepsis-Induced Cardiomyopathy: a Comprehensive Review. *Curr. Cardiol. Rep.* 22:35. 10.1007/s11886-020-01277-2 PMC7222131 32377972

[B15] Li F. Luo J. Wu Z. Xiao T. Zeng O. Li L. (2016). Hydrogen sulfide exhibits cardioprotective effects by decreasing endoplasmic reticulum stress in a diabetic cardiomyopathy rat model. *Mol. Med. Rep.* 14 865–873. 10.3892/mmr.2016.5289 27222111

[B16] Li N. Zhou H. Wu H. Wu Q. Duan M. Deng W. (2019). STING-IRF3 contributes to lipopolysaccharide-induced cardiac dysfunction, inflammation, apoptosis and pyroptosis by activating NLRP3. *Redox Biol.* 24:101215. 10.1016/j.redox.2019.101215 31121492 PMC6529775

[B17] Li X. Cheng Q. Li J. He Y. Tian P. Xu C. (2017). Significance of hydrogen sulfide in sepsis-induced myocardial injury in rats. *Exp. Ther. Med.* 14 2153–2161. 10.3892/etm.2017.4742 28962136 PMC5609143

[B18] Ling Q. Yu X. Wang T. Wang S. G. Ye Z. Q. Liu J. H. (2017). Roles of the Exogenous H_2_S-Mediated SR-A Signaling Pathway in Renal Ischemia/Reperfusion Injury in Regulating Endoplasmic Reticulum Stress-Induced Autophagy in a Rat Model. *Cell Physiol. Biochem.* 41 2461–2474. 10.1159/000475915 28472786

[B19] Liu J. Li J. Tian P. Guli B. Weng G. Li L. (2019). H_2_S attenuates sepsis-induced cardiac dysfunction via a PI3K/Akt-dependent mechanism. *Exp. Ther. Med.* 17 4064–4072. 31007743 10.3892/etm.2019.7440 PMC6468938

[B20] Liu L. Wu H. Zang J. Yang G. Zhu Y. Wu Y. (2016). 4-Phenylbutyric Acid Reveals Good Beneficial Effects on Vital Organ Function via Anti-Endoplasmic Reticulum Stress in Septic Rats. *Crit. Care Med.* 44 e689–e701. 26958745 10.1097/CCM.0000000000001662

[B21] Liu Y. C. Yu M. M. Shou S. T. Chai Y. F. (2017). Sepsis-Induced Cardiomyopathy: Mechanisms and Treatments. *Front. Immunol.* 8:1021. 10.3389/fimmu.2017.01021 PMC5609588 28970829

[B22] Miao L. Shen X. Whiteman M. Xin H. Shen Y. Xin X. (2016). Hydrogen Sulfide Mitigates Myocardial Infarction via Promotion of Mitochondrial Biogenesis-Dependent M2 Polarization of Macrophages. *Antioxid Redox Signal* 25 268–281. 10.1089/ars.2015.6577 27296720

[B23] Ndongson-Dongmo B. Heller R. Hoyer D. Brodhun M. Bauer M. Winning J. (2015). Phosphoinositide 3-kinase gamma controls inflammation-induced myocardial depression via sequential cAMP and iNOS signalling. *Cardiovasc. Res.* 108 243–253. 10.1093/cvr/cvv217 26334033

[B24] Pang J. Peng H. Wang S. Xu X. Xu F. Wang Q. (2019). Mitochondrial ALDH2 protects against lipopolysaccharide-induced myocardial contractile dysfunction by suppression of ER stress and autophagy. *Biochim. Biophys. Acta Mol. Basis Dis.* 1865 1627–1641. 10.1016/j.bbadis.2019.03.015 30946956

[B25] Parker M. M. Shelhamer J. H. Bacharach S. L. Green M. V. Natanson C. Frederick T. M. (1984). Profound but reversible myocardial depression in patients with septic shock. *Ann. Intern. Med.* 100 483–490. 10.7326/0003-4819-100-4-483 6703540

[B26] Perridon B. W. Leuvenink H. G. Hillebrands J. L. van Goor H. Bos E. M. (2016). The role of hydrogen sulfide in aging and age-related pathologies. *Aging* 8 2264–2289. 10.18632/aging.101026 27683311 PMC5115888

[B27] Singer M. Deutschman C. S. Seymour C. W. Shankar-Hari M. Annane D. Bauer M. (2016). The Third International Consensus Definitions for Sepsis and Septic Shock (Sepsis-3). *JAMA* 315 801–810. 26903338 10.1001/jama.2016.0287 PMC4968574

[B28] Sodhi C. P. Jia H. Yamaguchi Y. Lu P. Good M. Egan C. (2015). Intestinal Epithelial TLR-4 Activation Is Required for the Development of Acute Lung Injury after Trauma/Hemorrhagic Shock via the Release of HMGB1 from the Gut. *J. Immunol.* 194 4931–4939. 10.4049/jimmunol.1402490 25862813 PMC4417407

[B29] Sun L. T. Chen L. Wang F. G. Zheng X. Yuan C. F. (2019). Exogenous hydrogen sulfide prevents lipopolysaccharide- induced inflammation by blocking the TLR4/NF-κB pathway in MAC-T cells. *Gene* 8 114–121. 10.1016/j.gene.2019.05.033 31153885

[B30] Tan B. Jin S. Sun J. P. Gu Z. K. Sun X. T. (2017). New method for quantification of gasotransmitter hydrogen sulfide in biological matrices by LC-MS/MS. *Sci. Rep.* 4:46278. 10.1038/srep46278 PMC5390247 28406238

[B31] Tan S. Long Z. Hou X. Lin Y. Xu J. You X. (2019). H_2_ Protects Against Lipopolysaccharide-Induced Cardiac Dysfunction via Blocking TLR4-Mediated Cytokines Expression. *Front. Pharmacol.* 10:865. 10.3389/fphar.2019.00865 PMC6694767 31440160

[B32] Tang J. Hu J. J. Lu C. H. (2014). Propofol inhibits lipopolysaccharide-induced tumor necrosis factor-alpha expression and myocardial depression through decreasing the generation of superoxide anion in cardiomyocytes. *Oxid Med. Cell Longev.* 2014:157376. 10.1155/2014/157376 PMC4144395 25180066

[B33] Wang S. Binder P. Fang Q. Wang Z. Xiao W. Liu W. (2018). Endoplasmic reticulum stress in the heart: insights into mechanisms and drug targets. *Br. J. Pharmacol.* 175 1293–1304. 10.1111/bph.13888 28548229 PMC5867005

[B34] Wu D. Hu Q. X. Zhu Y. Z. (2016). Therapeutic application of hydrogen sulfide donors: the potential and challenges. *Front. Med.* 10:18–27. 10.1007/s11684-015-0427-6 26597301

[B35] Xia H. Li Z. Sharp T. E. III Polhemus D. J. Carnal J. Moles K. H. (2020). Endothelial Cell Cystathionine γ-Lyase Expression Level Modulates Exercise Capacity, Vascular Function, and Myocardial Ischemia Reperfusion Injury. *J. Am. Heart Assoc.* 9:e017544. 10.1161/JAHA.120.017544 PMC7792404 32990120

[B36] Yang F. M. Zuo Y. Zhou W. Xia C. Hahm B. Sullivan M. (2018). sNASP inhibits TLR signaling to regulate immune response in sepsis. *J. Clin. Invest.* 128 2459–2472. 10.1172/jci95720 29733298 PMC5983344

[B37] Yin P. Zhao C. Li Z. Mei C. Yao W. (2012). *Sp1* is involved in regulation of cystathionine gamma-lyase gene expression and biological function by PI3K/Akt pathway in human hepatocellular carcinoma cell lines. *Cell Signal* 24 1229–1240. 10.1016/j.cellsig.2012.02.003 22360859

[B38] Yuan S. Shen X. Kevil C. G. (2017). Beyond a Gasotransmitter: Hydrogen Sulfide and Polysulfide in Cardiovascular Health and Immune Response. *Antioxid Redox Signal* 27 634–653. 10.1089/ars.2017.7096 28398086 PMC5576200

[B39] Zhang B. Liu Y. Zhang J. S. Zhang X. H. Chen W. J. Yin X. H. (2015). Cortistatin protects myocardium from endoplasmic reticulum stress induced apoptosis during sepsis. *Mol. Cell Endocrinol.* 406 40–48. 10.1016/j.mce.2015.02.016 25727193

[B40] Zhang H. X. Du J. M. Ding Z. N. Zhu X. Y. Jiang L. Liu Y. J. (2017). Hydrogen sulfide prevents diaphragm weakness in cecal ligation puncture-induced sepsis by preservation of mitochondrial function. *Am. J. Transl. Res.* 9 3270–3281. 28804545 PMC5553877

[B41] Zhang H. Zhi L. Moore P. K. Bhatia M. (2006). Role of hydrogen sulfide in cecal ligation and puncture-induced sepsis in the mouse. *Am. J. Physiol. Lung Cell Mol. Physiol.* 290 L1193–L1201. 16428267 10.1152/ajplung.00489.2005

[B42] Zhang Z. Jin S. Teng X. Duan X. Chen Y. Wu Y. (2017). Hydrogen sulfide attenuates cardiac injury in takotsubo cardiomyopathy by alleviating oxidative stress. *Nitric Oxide* 67 10–25. 10.1016/j.niox.2017.04.010 28450188

[B43] Zhao H. Zhang M. Zhou F. Cao W. Bi L. Xie Y. (2016). Cinnamaldehyde ameliorates LPS-induced cardiac dysfunction via TLR4-NOX4 pathway: The regulation of autophagy and ROS production. *J. Mol. Cell Cardiol.* 101 11–24. 10.1016/j.yjmcc.2016.10.017 27838370

[B44] Zhong J. Tan Y. Lu J. Liu J. Xiao X. Zhu P. (2019). Therapeutic contribution of melatonin to the treatment of septic cardiomyopathy: A novel mechanism linking Ripk3-modified mitochondrial performance and endoplasmic reticulum function. *Redox Biol.* 26:101287. 10.1016/j.redox.2019.101287 31386965 PMC6692063

