# Supplementary material for: Hydrogen Sulfide Attenuated Sepsis-Induced Myocardial Dysfunction Through TLR4 Pathway and Endoplasmic Reticulum Stress
Source: Front Physiol. 2021 Jun 9;12:653601. doi: 10.3389/fphys.2021.653601 (PMC8220204; doi:10.3389/fphys.2021.653601)
Supplement: Supplementary file 1 [file Data_Sheet_1.docx]

**Supplementary Figure 1**

**
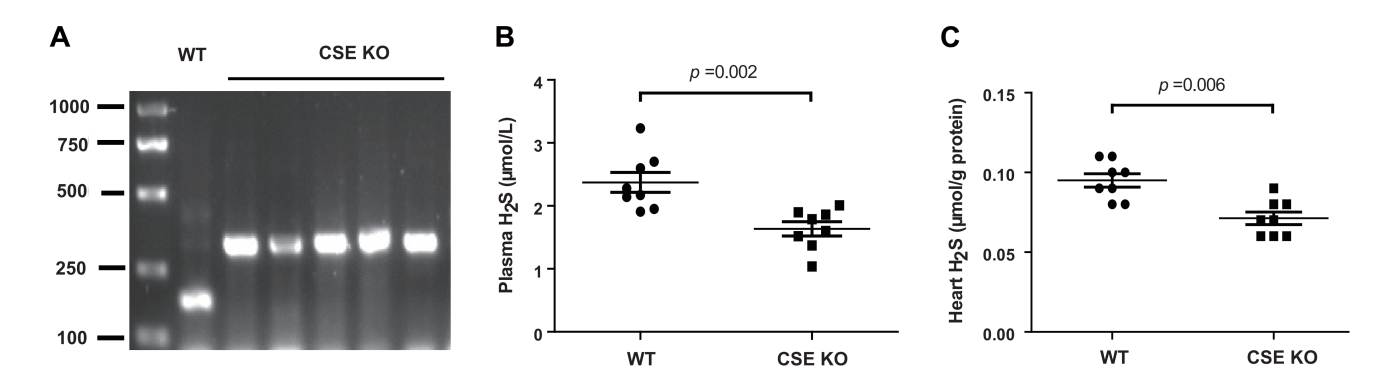
**

**Supplementary Figure 1 Characterization of CSE KO mice: (A)**Representative PCR images of CSE gene in the genomic DNA from WT mice and CSE KO mice. **(B)** H_2_S levels in the plasma. **(C)** H_2_S levels in the heart tissues. n=8 in every group. Results are means ± SEM. *p*< 0.05 was considered significant.

**Supplementary Figure 2**

**
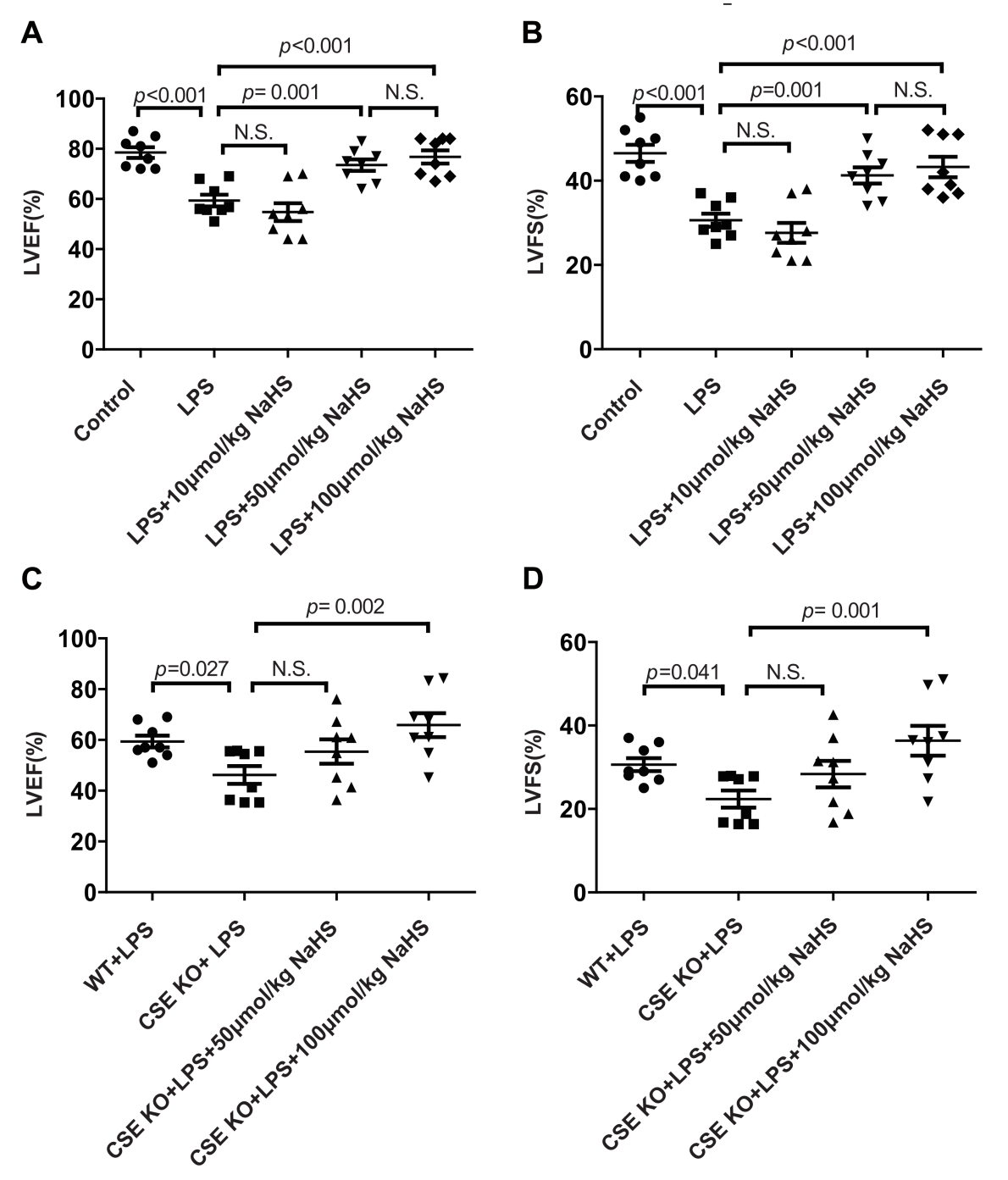
**

**Supplementary Figure 2 Echocardiographic parameters of different do****ses of NaHS on cardiac function: (A and B)** The C57BL/6J mice received different doses of NaHS (10, 50, 100µmol/kg) intraperitoneally 3h after administration of LPS (10 mg/kg) intraperitoneally. The changes of LVEF and LVFS in each group. **(C and D)** The CSE KO mice received different doses of NaHS (50, 100µmol/kg) intraperitoneally 3h after administration of LPS (10 mg/kg) intraperitoneally. The changes of LVEF and LVFS in each group. n=8 in every group. Results are means ± SEM. *p*< 0.05 was considered significant.
